# A 36-Year-Old Female With Congenital Contractural Arachnodactyly and Pectus Excavatum Requiring Fourth-Time Redo Surgical Correction

**DOI:** 10.7759/cureus.16701

**Published:** 2021-07-28

**Authors:** Rachel S Dada, Jeremiah W Hayanga, Mir Ali Abbas Khan, Alper Toker, Heather K Hayanga

**Affiliations:** 1 Department of Anesthesiology, West Virginia University, Morgantown, USA; 2 Department of Cardiovascular and Thoracic Surgery, West Virginia University, Morgantown, USA; 3 Department of Cardiovascular and Thoracic Anesthesiology, West Virginia University, Morgantown, USA

**Keywords:** beal's syndrome, congenital contractural arachnodactyly, pectus excavatum, nuss procedure, thoracic surgery, cardiac anesthesia, echocardiography, cardiac physiology

## Abstract

Congenital contractural arachnodactyly (CCA) is a rare connective tissue disorder that has several phenotypic similarities to Marfan syndrome. Among the phenotypic characteristics of patients with CCA, severe kyphoscoliosis and thoracic cage abnormalities are commonly reported. In this case report, we describe a patient with coexisting CCA and severe pectus excavatum requiring multiple surgical repairs. The impact severe scoliosis and pectus excavatum in isolation have on cardiopulmonary anatomy and physiology can be significant, and their effects can be profound concomitantly. These defects have the propensity of causing restrictive lung disease and external cardiac compression.

## Introduction

Congenital contractural arachnodactyly (CCA), also known as Beals Syndrome, is a rare genetic connective tissue disorder of autosomal-dominant inheritance caused by a mutation of the Fibrillin-2 gene (FBN2) on chromosome 5q23 [1]. Phenotypic characteristics of patients with CCA are exceedingly similar to that of Marfan Syndrome and include multiple flexion contractures, arachnodactyly, severe kyphoscoliosis, crumpled pinnae, and muscular hypoplasia [1,2]. Thoracic cage abnormalities may also be present potentially causing restrictive lung disease [1,2].

Pectus deformities have been observed in association with scoliosis and connective tissue disorders [1,3]. Pectus excavatum alone has the propensity to negatively impact cardiopulmonary function which can be manifested clinically with exercise intolerance, chest pain, shortness of breath, and palpitations [3]. 

## Case presentation

**Figure 1:**
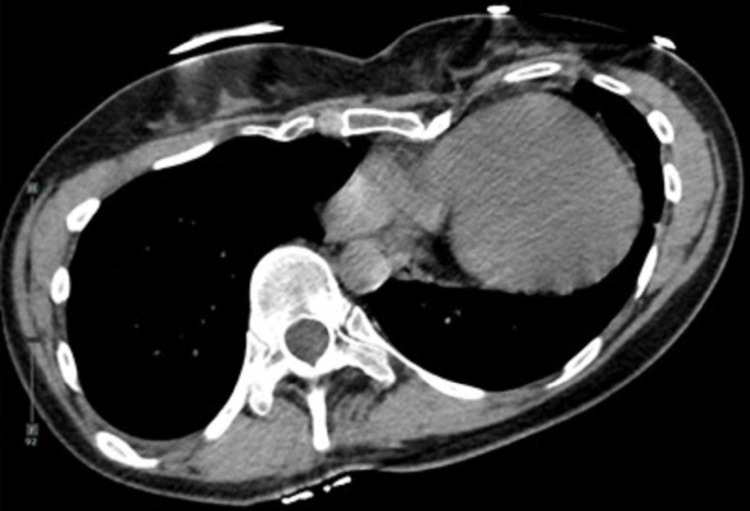
Preoperative chest computed tomography showing significant pectus deformity causing leftward cardiac displacement and direct cardiac compression with subsequent left lung compression.

**Figure 2:**
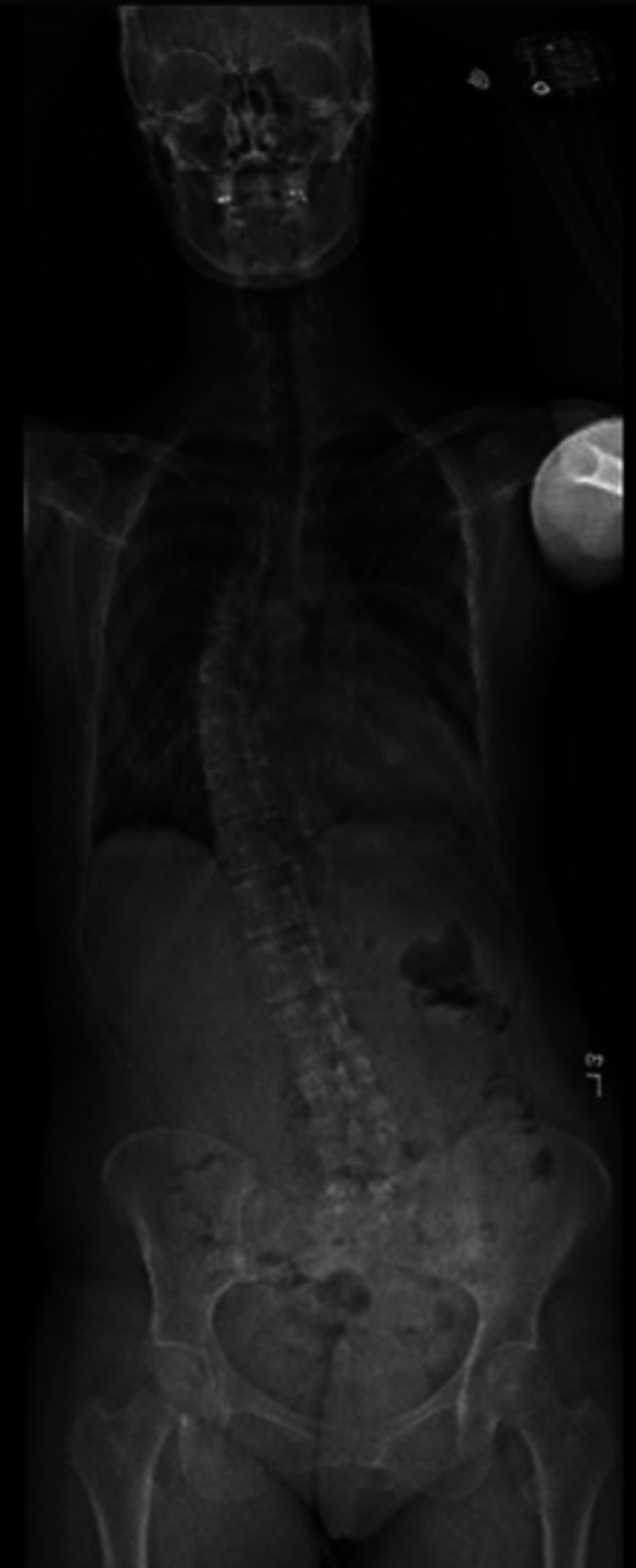
40° thoracolumbar dextroscoliosis

A 36-year-old female with CCA and pectus excavatum underwent two pediatric Ravitch procedures and as a 30-year-old she underwent a modified Ravitch procedure with excision of bilateral second rib cartilages. She initially had improvement in shortness of breath, but presented six years later with worsening dyspnea, exertional chest pain, and significant structural deformity. Preoperative evaluation included pulmonary function testing (PFTs), chest computed tomography (CT), transthoracic echocardiography (TTE), and scoliosis radiographs. PFTs revealed moderate restrictive lung impairment while chest CT was significant for a Haller index greater than 6, external cardiac compression, and leftward cardiac displacement within the thoracic cavity (Figure 1). TTE demonstrated left ventricle (LV) ejection fraction (EF) of 50-55% with external compression of the right atrium and basal right ventricle (RV) noted and trace tricuspid regurgitation, and scoliosis radiographs were significant for 40° thoracolumbar dextroscoliosis (Figure 2). She underwent neurosurgical evaluation of her scoliosis, and no curvature progression was noted. As a result, she was deemed to not require surgical correction of her scoliosis.

**Figure 3:**
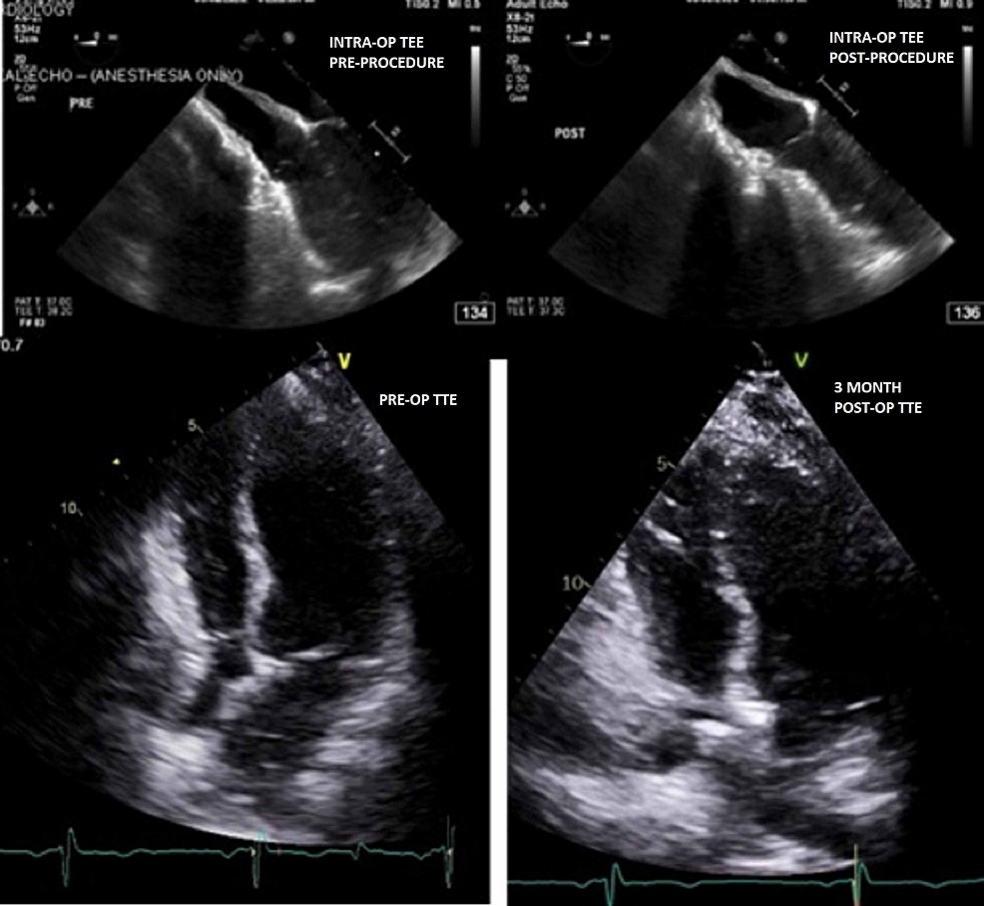
Intraoperative transesophageal echocardiography (top): Modified mid-esophageal 4-chamber view pre- (left) and post-procedure (right) Transthoracic echocardiography (bottom): Preoperative (left) and 3 months postoperative (right)

Given her symptoms and imaging findings, she was scheduled for reoperation. The surgery was thoroughly planned with detailed evaluations and imaging used to determine incision selection, areas for reconstruction, and cartilage excision. The thoracic surgeons determined that an open reconstruction of the chest wall using a Nuss Strut would be most appropriate. Preoperatively, bilateral erector spinae plane catheters were placed. Due to concern for potential cardiopulmonary compromise from cardiac compression or mediastinal shift, defibrillator pads were placed on the patient prior to induction of anesthesia. General anesthesia was gradually induced using a combination of midazolam, lidocaine, ketamine, and propofol with preservation of spontaneous ventilation until it was observed that the patient had preserved hemodynamic and respiratory stability. Paralysis was subsequently achieved with succinylcholine to facilitate intubation, followed by rocuronium for continued paralysis. Because the surgical team performed the procedure via an open approach, two-lung ventilation throughout the procedure was considered to be acceptable, so endotracheal intubation was performed utilizing a single-lumen endotracheal tube. Following endotracheal intubation, a volume-controlled mode of ventilation was selected in an effort to minimize airway pressures while simultaneously achieving adequate tidal volume. Peak inspiratory pressures were consistently <20 cmH_2_O throughout the procedure while continually achieving tidal volumes of approximately 7-8 mL/kg ideal body weight. An 8-French double-lumen central venous catheter and an arterial line were placed in addition to transesophageal echocardiography (TEE) to guide intraoperative management. Baseline intraoperative TEE noted LV EF of 60-65%, redemonstration of external compression of the right atrium and basal right ventricle, no tricuspid regurgitation while the pulmonic valve was not well-visualized. She underwent an open reconstruction of the chest wall using a Nuss Strut, partial vertical splitting of the lower sternum, resection of left lower cartilages, and reconstruction with Gore-Tex mesh. The patient remained hemodynamically stable throughout the duration of the procedure, with an episode of mild hypotension upon insertion of the Nuss bar which responded to fluid administration. Both intraoperative TEE and postoperative TTE noted interval increase in right atrial and ventricular cavity sizes with normal RV function (Figure 3). Left ventricular function improved slightly from 50-55% to 68%. Despite the notable changes observed on the TEE, no significant changes involving the patient’s pulmonary status were seen, as peak inspiratory pressures remained consistently <20 cmH_2_O both pre- and post- procedure.

**Figure 4:**
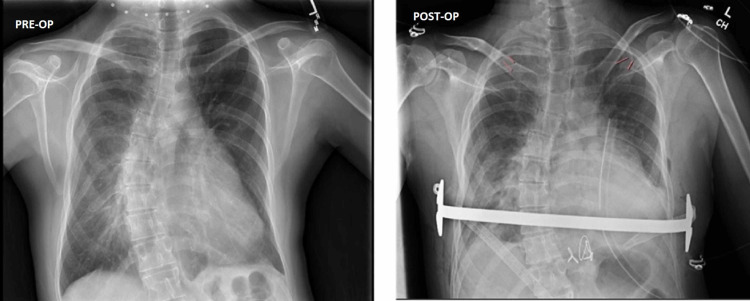
Preoperative (left) and postoperative (right) chest radiographs

The goal of extubation in the operating room following the case was set preoperatively. Therefore, a heavy emphasis was placed on multi-modal analgesia in an effort to minimize opioid-induced respiratory depression, placing this patient at risk for requiring reintubation. In addition to the preoperatively placed erector spinae plane blocks, intraoperative multimodal analgesia was provided by utilizing a combination of acetaminophen, diazepam, dexmedetomidine, ketamine, hydromorphone, and fentanyl. The patient was successfully extubated in the operating room at the end of the case and taken to recovery in stable condition. Postoperative pain control was achieved by continuing the patient’s pregabalin, bilateral erector spinae plane catheters running lidocaine infusions which were continued for five days, a ketamine infusion, and hydromorphone patient-controlled analgesia (PCA) that was subsequently converted to oral hydromorphone and scheduled acetaminophen. Postoperative chest radiograph revealed postoperative changes for treatment of pectus excavatum with fluid and airspace opacity of the left lung base compared to preoperative evaluation (Figure 4).

The patient’s postoperative course was initially complicated by pain and discomfort lasting nearly six weeks after surgery, however, this improved significantly with the aforementioned management and time. Two months postoperatively, the patient was able to appreciate the marked improvement in her exertional chest pain and dyspnea. Approximately three months after surgery, the patient presented to the emergency department with complaints of a painful, pruritic rash overlying the insertion site of the metal bar. This was concerning for an allergic reaction to the metal implant, an event that has been reported to occur in approximately 2.7% of patients following a Nuss procedure [4]. Skin biopsy was significant for spongiotic dermatitis, confirming the diagnosis of an allergic reaction to the metal bar. At this time, the surgical team is working with the biomedical engineering department at our institution to potentially develop a bar developed from non-metallic material if the patient’s allergy becomes unforgiving.

## Discussion

Congenital contractural arachnodactyly is a genetic connective tissue disorder that involves a defect in the FBN2 gene with resultant decreased production and/or impaired function of fibrillin-2 [5]. Fibrillin-2 has been implicated in the regulation of local transforming growth factor-beta (TGF-beta) and bone morphogenetic protein (BMP), both of which are potent modulators of extracellular matrix formation and remodeling [6]. Osteoblasts deficient in fibrillin-2 have been found to exhibit impaired bone formation with improper maturation, as fibrillin-2 expression plays an important role in bone mechanical properties and integrity [6,7]. Arteaga-Solis et al. determined that the femurs of mice lacking the FBN2 gene have significantly lower strength index, maximum load, and stiffness when compared to wild-type bones [7].

Due to the phenotypic overlap of CCA with Marfan syndrome, recognition of CCA as a separate entity has been historically challenging. Despite advances in medicine and technology, diagnosis of CCA remains difficult. In 1971, CCA was first differentiated from Marfan Syndrome by Beals and Hecht who identified in the literature 12 kindreds with characteristics suggestive of CCA [2]. Following the discovery of the FBN2 gene mutation, the diagnosis of CCA has been reported with increased frequency, although true prevalence remains unknown [1]. Despite advancements in genetic testing, CCA can still be challenging to diagnose. At present, the diagnosis of CCA can be made in the presence of a heterozygous FBN2 pathologic variant as well as suggestive phenotypic findings [8]. However, not all individuals with CCA have an identifiable FBN2 pathologic variant due to locus heterogeneity. Thus, Meerschaut et al. developed a novel clinical scoring system for patients with suspected CCA but without a pathogenic FBN2 variant, intellectual disability, ectopia lentis, and/or progressive aortic root dilatation. In this scoring system, scores of ≥7/20 and ≥11/20 carry 95.5% and 75% sensitivity, respectively, and 17.1% and 60% specificity (Table 1) [8,9].

**Table 1:** A clinical scoring system for CCA. Clinical scoring system based on Callewaert (2019) [7]. CAA: congenital contractural arachnodactyly

Clinical Feature	Points	Notes
Arachnodactyly	3	Assessed by evaluating the thumb and wrist signs
Camptodactyly	3	
Crumpled ears	3	In a neonate: often demonstrates folding and incomplete development of the upper portion of the helix, a prominent helical & inferior crus of the antihelix In older children/adults: while the ear may become less folded, the crura prominence remains
Large-joint contractures	3	
Dolichostenomelia	2	Presence of decreased US/LS ratio (<0.85 white adults, <0.78 black adults) AND increased arm-span-to-height ratio (>1.05) without significant scoliosis
Pectus deformity	2	
Highly arched palate	1	
Kyphoscoliosis	1	Clinical or radiographic diagnosis
Micrognathia	1	
Muscle hypoplasia	1	

In patients identified with CCA, congenital cardiac defects have been detected in up to 32.2%, though they are most commonly asymptomatic atrial or ventricular septal defects or mitral valve prolapse [10]. Clinically significant cardiac defects of CCA are less frequently observed than in Marfan syndrome but can include interrupted aortic arch and aortic root dilation [8,10]. Although cardiac defects may occur, cardiorespiratory symptoms that develop in patients with CCA are often due to the sequelae of thoracic cage and chest wall deformities resulting in restrictive pulmonary disease and cardiac compression [3]. 

The incidence of kyphoscoliosis in CCA is 62%, and it may occur congenitally or develop and/or worsen during periods of fast growth [8]. In their 2014 study, Huh et al. found significant negative correlations between the Cobb angle and forced vital capacity (FVC), forced expiratory volume in one second (FEV1), mitral E/A ratio, and tissue Doppler E’/A’ values [11]. The Cobb angle, a radiographically obtained measurement, quantifies the degree of scoliosis by measuring the angle between intersecting lines which are perpendicular to the top and bottom of the most tilted vertebrae above and below the apex of the curve, respectively [11]. 

Our patient not only had CCA with kyphoscoliosis, but she also had pectus excavatum. The incidence of pectus deformity in patients with CCA is 41% and is a result of rib overgrowth with subsequent inward or outward deviation of the sternum and anterior thoracic wall [8]. The pectus deformity can thus be excavatum or carinatum; however, excavatum is more common [8]. The Haller index, a ratio of thoracic width and height, is the gold standard for depicting the severity of chest wall deformity in pectus excavatum [12]. A Haller index score of 2.5-2.7 is considered to be normal, while a Haller index of ≥3.25 is considered to be severe [3]. Although the Haller index is used as a marker of pectus excavatum severity, symptomatic presentation can be variable [13]. In their 2012 study, Swanson et al. concluded that a Haller index of >3.6 was associated with pulmonary dysfunction [14]. Additionally, pectus excavatum also has the propensity to cause direct cardiac compression and deviation [3]. Cardiac symptomatology that develops as a result of pectus excavatum relates directly to the reduced sternovertebral distance [13]. The compromised sternovertebral distance and compensatory leftward cardiac rotation and displacement leave the right ventricle particularly susceptible to direct compression from the depressed sternum [13]. The physiological impact of pectus excavatum on the heart can be significant, as right ventricular compression and impaired filling can result in a net decrease in cardiac output and stroke volume [15]. 

Studies have investigated the impact surgical repair of pectus excavatum has on the thoracic spine in the setting of coexisting thoracic scoliosis and have demonstrated improvement in scoliosis for patients with a lower preoperative Cobb angle as well as younger patients [16]. Surgical correction of pectus excavatum can positively impact cardiac function due to relief of direct cardiac compression allowing for increased right ventricular distensibility and improved stroke volume and cardiac output [15,17]. In an effort to maintain adequate cardiac output despite the reduced stroke volume, patients with severe pectus excavatum experience compensatory tachycardia to maintain adequate cardiac output [17]. In addition, increased sternovertebral distance following surgery facilitates improvement in the restrictive lung disease caused by direct compression [15]. Surgical expansion of the thoracic cavity allows for increased capacity to generate negative thoracic pressure with inspiration leading to improved right-sided cardiac filling [15]. Studies have also found improvement in post-surgical RV function, including increased RV end-diastolic diameter, RV end-diastolic volume index, RV systolic function, and increased RV chamber size [15,17,18].

Although multiple studies have demonstrated improvement in overall cardiac function following pectus excavatum repair, left ventricular ejection fraction has not been found to consistently significantly increase following surgery [17,19,20]. As a result of this, Gürkan et al. also evaluated LV myocardial performance index in their study [17]. Despite the absence of significant LV EF improvement, they found significant improvement in LV myocardial performance index (LV Tei index) following pectus excavatum repair. The LV Tei index is more sensitive than LV EF at detecting improvements in both systolic and diastolic LV function [17]. Left ventricular EF may remain constant as pre-surgical LV preload is low due to low RV preload from impaired filling due to compression and suboptimal generation of negative intrathoracic pressure due to restrictive lung disease. This may be explained by the improved cardiac output and stroke volume following surgery [15,17].

## Conclusions

In isolation, both CCA and pectus excavatum may negatively impact cardiopulmonary status and function. In combination, it can be compounded. Surgical repair of the pectus deformity not only can lead to desirable cosmetic effects, but can also relieve cardiac compression and thoracic restriction, thereby improving cardiorespiratory symptoms.
